# Musical experience prior to traumatic exposure as a resilience factor: a conceptual analysis

**DOI:** 10.3389/fpsyg.2023.1220489

**Published:** 2023-07-27

**Authors:** Elodie Fraile, Pierre Gagnepain, Francis Eustache, Mathilde Groussard, Hervé Platel

**Affiliations:** Neuropsychology and Imaging of Human Memory (NIMH) Research Unit, PSL Research University-EPHE-INSERM-Caen University Hospital-Cyceron-Normandy University, Caen, France

**Keywords:** music, resilience, reserve, pretraumatic, posttraumatic stress disorder (PTSD), prevention, emotion regulation, cognitive control

## Abstract

Resilience mechanisms can be dynamically triggered throughout the lifecourse by resilience factors in order to prevent individuals from developing stress-related pathologies such as posttraumatic stress disorder (PTSD). Some interventional studies have suggested that listening to music and musical practice *after* experiencing a traumatic event decrease the intensity of PTSD, but surprisingly, no study to our knowledge has explored musical experience as a potential resilience factor *before* the potential occurrence of a traumatic event. In the present conceptual analysis, we sought to summarize what is known about the concept of resilience and how musical experience could trigger two key mechanisms altered in PTSD: emotion regulation and cognitive control. Our hypothesis is that the stimulation of these two mechanisms by musical experience during the pre-traumatic period could help protect against the symptoms of emotional dysregulation and intrusions present in PTSD. We then developed a new framework to guide future research aimed at isolating and investigating the protective role of musical experience regarding the development of PTSD in response to trauma. The clinical application of this type of research could be to develop pre-trauma training that promotes emotional regulation and cognitive control, aimed at populations at risk of developing PTSD such as healthcare workers, police officers, and military staffs.

## 1. Introduction

Based on responses to WHO World Mental Health Surveys, it has been estimated that more than 7 out of 10 people worldwide have been exposed to psychological trauma (Kessler et al., 2017). Major categories of trauma include war-related trauma, physical or sexual violence, accidents, and the unexpected death of a loved one (Kessler et al., 2017). Of those who have been exposed to trauma, 5.6% go on to develop posttraumatic stress disorder (PTSD), according to a cross-national study (Koenen et al., 2017). The pathogenesis of PTSD is explained in particular by stress-induced structural and functional impairments in emotion and memory circuits (Bremner, 2006; Shin, 2006; Francati et al., 2007; Dégeilh et al., 2013; Postel et al., 2019; Harnett et al., 2020). Impairment of prefrontal mechanisms that regulate activity in emotion (Kaldewaij et al., 2021) and memory (Mary et al., 2020; Leone et al., 2022) circuits, and which normally act upon the negative effect of stress, has also been identified as contributing to the development of PTSD. As a consequence, PTSD has been characterized as a failure to deploy the adaptation processes needed to recover from the normal effects of trauma and promote resilience (Yehuda and LeDoux, 2007; Kalisch et al., 2017). Not all individuals exposed to trauma exhibit stress-induced modifications of emotion and memory circuits. It is therefore crucial to understand the factors and mechanisms that may explain interindividual differences and enable individuals to be resilient.

*Resilience* can be defined as resulting from “a dynamic process of adaptation to the given stressful life circumstances” (Kalisch et al., 2017, p. 2). This process includes resilience factors and resilience mechanisms for dealing with stress-induced alterations (Kalisch et al., 2017; Pascual-Leone and Bartres-Faz, 2021). Resilience factors are stable characteristics or predispositions that facilitate the activation of resilience mechanisms in a response to a stressor, in order to prevent individuals from developing stress-related pathologies (Kalisch et al., 2017). They can be biological, social, or environmental. Environmental factors are among the easiest to modify (von Haumeder et al., 2019). More specifically, participation in cultural and artistic activities, including playing and listening to music, is often consider a resilience factor (Matziorinis et al., 2022). While reactions to a stressor depend on both pre-and post-traumatic resilience factors (Kalisch et al., 2017), most studies have explored the benefits of musical activity *after* the occurrence of a traumatic event as an intervention tool to reduce the intensity of PTSD, and not *before* as a preventive tool against the deleterious effects of potential exposure to a traumatic situation (Landis-Shack et al., 2017; Pant et al., 2022).

Playing and listening to music activates mechanisms that may be relevant in the treatment of PTSD (Pant et al., 2022), but the impact of musical experience prior to the trauma as a factor for resilience remains underexplored. This is an area worth studying insofar as the benefits of musical activity promote emotion regulation (Moore, 2013) and inhibitory control resulting in greater resistance to interference (Bialystok and DePape, 2009; Moreno and Farzan, 2015; Moussard et al., 2016; D’Souza et al., 2018). As resilience is a dynamic process linked to the preservation of effective plasticity mechanisms, and as musical experience induces strong neuroplasticity, it seems relevant to ask whether a lifestyle with a strong emphasis on music might be a pre-traumatic resilience factor (Jäncke, 2009; Herholz and Zatorre, 2012; Schlaug, 2015).

The purpose of the present conceptual analysis is to determine whether listening to music and musical practice have protective effects against the development of PTSD, and limit PTSD-related cognitive and brain impairments by enhancing resilience. In other words, the aim of this paper is not to present exhaustively contributions or methodological weaknesses of the work on music therapy and PTSD already documented in the literature (Landis-Shack et al., 2017; Pant et al., 2022), but to focus on some mechanisms triggered by musical experience that could be protective in the event of exposure to trauma. A description of the morphological and neurofunctional bases of PTSD and resilience is followed by a proposal for future research directions, focusing on the interaction between musical experience, resilience processes, and the emotion regulation (ER) and cognitive control mechanisms that are central to the development of PTSD.

## 2. Neurofunctional bases of trauma, PTSD, and resilience

### 2.1. Definition of trauma and the impact of stress on the brain

Stress has a neurotoxic effect on several brain regions, including three brain regions that are usually involved in regulating the harmful effects of stress: the prefrontal cortex (PFC), amygdala, and hippocampus (Bremner, 2006; McEwen, 2017; Kaul et al., 2021). All three brain regions are modified in PTSD.

The PFC is associated with the executive functions that allow us to adapt to new or nonroutine situations (Roberts et al., 1998; Miller and D’Esposito, 2005; Euston et al., 2012). Executive functions include a range of mechanisms, not least cognitive control and inhibitory control (Cristofori et al., 2019). *Cognitive control* refers to processes that allow us to adapt “to achieve a given goal in a given situation” (Miller, 2000, p. 1). *Inhibitory control* (*inhibition*) is used to suppress inappropriate responses (Miyake et al., 2000; Diamond, 2013) or interfering activities, such as intrusive memories (Gagnepain et al., 2017b; Anderson and Hulbert, 2021). Chronic stress causes the dendrites of neurons in the medial PFC (mPFC) to shorten and debranch. This has harmful effects on inhibitory functions, giving rise to cognitive rigidity and task-switching difficulties (McEwen et al., 2015; Morris and Mansell, 2018). Functional and structural modifications of the PFC are observed in PTSD (Lanius et al., 2006; Shin, 2006). At the anatomical level, atrophy of the mPFC has been reported in numerous studies, while at the functional level, hypoactivation of the mPFC has been observed in patients with PTSD (Shin, 2006; Harnett et al., 2020), as well as aberrant connectivity between frontal and memory regions during memory control (Mary et al., 2020; Leone et al., 2022).

The amygdala, another structure impacted by stress, is essential for orienting our reactions to perceived emotions. This warning system plays a central role in fear conditioning and in the emotional modulation of memory (Davis and Whalen, 2001; Phelps and LeDoux, 2005; Ehrlich et al., 2009). The top-down control of the amygdala by the mPFC modulates fear reactions (Marek et al., 2019). The effects of stress on the structure of the amygdala vary according to the type and chronicity of that stress (Zhang et al., 2021). Although no structural changes in the amygdala have been specifically observed in PTSD, functional neuroimaging studies using functional MRI (fMRI) and PET techniques have consistently revealed hyperactivation of the amygdala (Shin, 2006; Hayes et al., 2012; Harnett et al., 2020). This hyperactivation disrupts the inhibition of fear and anxiety, thereby hindering ER (Milad et al., 2009; Andrewes and Jenkins, 2019). *ER* can be defined as a mechanism “through which a person maintains a comfortable state of arousal by modulating one or more aspects of emotion” (Moore, 2013, p. 1) involving interaction between frontal control regions and areas involved in emotional reactivity, including the amygdala (Banks et al., 2007; Moore, 2013; Andrewes and Jenkins, 2019).

Finally, the hippocampus is often reported to be altered during exposure to stress. This structure plays a central role in the memory process, especially during the encoding and retrieval of information in episodic memory, as well as the re-experiencing of episodic details from the past (Tulving and Markowitsch, 1998; Viard et al., 2007; Piolino et al., 2009). Stress has toxic effects on the hippocampus, as it causes dendritic shortening and spine loss in the hippocampus (McEwen et al., 2015). In PTSD, no clear pattern has emerged concerning the functioning of the hippocampus. At the structural level, hippocampal atrophy has been observed in patients with PTSD, but there is no consensus yet over whether the atrophy is related to stress or to a pre-existing lower hippocampal volume (i.e., risk/vulnerability factor) (Geuze et al., 2005; Yehuda and LeDoux, 2007; Woon and Hedges, 2008; Logue et al., 2018; Postel et al., 2021). These brain alterations are consistent with the main symptoms of PTSD, although they may be explained by different pathophysiological processes.

### 2.2. Pathophysiological processes of PTSD

Four main symptoms are characteristic of PTSD according to the DSM-5 (American Psychiatric Association, 2013): (1) *reexperiencing* of sensations related to a past traumatic event in the form of intrusive memories, nightmares or flashbacks; (2) *avoidance* of experiencing or talking about situations that may evoke the traumatic event; (3) *thought and mood alteration*, including the inability to recall elements of the traumatic situation and a persistent negative emotional state; and (4) *disturbance of arousal and neurovegetative activity*. In addition, PTSD can be accompanied by dissociative symptoms. In the case of dissociation, states of depersonalization (feeling of detachment from self) or derealization (impression that the environment is unreal) can be experienced. The presence and intensity of PTSD symptoms may vary according to both the individual and the circumstances of the traumatic event, but the DSM-5 states that these four key symptoms must have lasted for more than 1 month following the traumatic event for a diagnosis of PTSD to be made (American Psychiatric Association, 2013). This symptom cluster reflects a complex and multidimensional pathology. Some aspects of PTSD have been explained by different cognitive and neurobiological complementary models, including theories that view PTSD as (1) a dysfunction of fear and threat circuits (Kredlow et al., 2022; Ressler et al., 2022), (2) as a memory disorder (see Brewin, 2018 for review) or (3) as a disorder of inhibitory control over emotion and memory (Andrewes and Jenkins, 2019; Mary et al., 2020; Leone et al., 2022). The research proposals made in this conceptual analysis focus on music as a means of stimulating emotional and memory regulation mechanisms prior to the onset of a potential traumatic event, with the aim of contributing to resilience mechanisms.

#### 2.2.1. PTSD as a dysfunction of fear and threat circuits

PTSD is a pathology that can be studied from the angle of dysregulation of normal fear and threats circuits (Ressler et al., 2022) leading to disturbance of neurovegetative activity and modifications of the state of arousal (Levy and Schiller, 2021; Kredlow et al., 2022). Regarding the disruption of threat response processes in PTSD, in normal circumstances, associations in the presence of a threat are (1) created, (2) learned, (3) stored, and then (4) updated. Individuals may also decide to (5) face the threat (Levy and Schiller, 2021). In the case of PTSD, however, one or more of these five steps are disturbed, owing to erroneous computations (Levy and Schiller, 2021). According to some authors, avoidance behaviors are linked to an inability to extinguish the initial fear and threat response (Ressler et al., 2022). According to others, however, because it is based on the anticipation of the threat, avoidance is linked to memory processes, insofar as the memorization of stimuli is likely to provoke flashbacks (Brewin, 2011).

#### 2.2.2. PTSD as a memory disorder

The focus here will be on a key memory-based model of PTSD, the dual representation theory originally formulated by Brewin et al. (1996) and revisited in 2010 (Brewin et al., 2010). According to this theory, memory disturbance is at the core of PTSD and intrusions arise from incongruence between contextual and sensory representations of memory (Brewin et al., 2010). Contextual memories refer to memory traces that can be retrieved consciously and voluntarily, while sensorial memories are retrieved involuntarily and independently of context. A situation of extreme fear and intense stress leads to a narrowing of the attentional focus on sensorial information, rather than on more elaborate cognitive activities. As a result, normal memory encoding is disrupted and contextual details are not properly processed. As the temporal context is not encoded, individuals process the information as if the threat were still present, hence the reexperiencing (e.g., flashbacks) (Brewin, 2011). In this case, impairment of the hippocampus, a key structure for memory, may be manifested by intrusions and/or disturbances in the encoding of contextual aspects of the traumatic event (Corcoran and Maren, 2001; Brewin et al., 2010).

#### 2.2.3. PTSD as a disorder of emotion and memory regulation mechanisms

Finally, an alternative and complementary approach suggest that PTSD is a failure of resilience mechanisms that normally allow for the regulation of emotions and memory (Marek et al., 2019; Anderson and Hulbert, 2021). In PTSD, PFC impairments may hinder the top-down regulation process of the PFC over structures such as amygdala and hippocampus, resulting in emotional and cognitive dysfunctions (Andrewes and Jenkins, 2019; Marek et al., 2019; Anderson and Hulbert, 2021). Longitudinal imaging studies have also shown an association between greater cortical thickness in the PFC and fewer manifestations of PTSD symptoms (Lyoo et al., 2011). Concerning emotions, the top-down control of the amygdala by the mPFC normally allows fear reactions to be modulated (Marek et al., 2019). But in PTSD the persistently negative emotional state can be explained by an inability of the mPFC to properly regulate amygdala hyperactivity, leading to emotional dysregulation (Andrewes and Jenkins, 2019). Emotional dysregulation can also result from structural and functional impairments of the anterior cingulate cortex, connected to both the PFC and the limbic system. This brain structure has reduced volume and is hypoactivated in PTSD, whereas it plays an important role in ER (Stevens et al., 2011; Fitzgerald et al., 2018; Dossi et al., 2020). Concerning intrusions, the influence of the PFC on the hippocampus could shed light on the mechanisms behind PTSD. *Active forgetting* mechanisms may reduce the accessibility of a memory through a process of memory inhibition, made possible by PFC control over on the hippocampus (Anderson and Hulbert, 2021). Impairments in inhibitory control regions of the lateral PFC or the gabaergic system, which executes inhibitory commands in the hippocampus (Schmitz et al., 2017), may disrupt the top-down regulation of the hippocampus supporting memory suppression, leading to intrusive memories (Mary et al., 2020; Anderson and Hulbert, 2021; Leone et al., 2022).

Stress-induced hyper-excitability is central to the development of traumatic memory and PTSD. Hence, mechanisms that support the gating of hyperexcitability and the flexible deactivation of fear and memory process, such as inhibitory control mechanisms, would be a possible general mechanism of resilience. Much research has been conducted on the factors that make individuals more vulnerable to trauma or stress-induced alterations in the brain. Beyond the stress response, however, little is known about the brain neural mechanisms that support resilience after trauma. Those resilience processes are yet equally important to understand PTSD (Yehuda and LeDoux, 2007). It is therefore crucial to articulate resilience research around neural mechanistic accounts describing the impact of resilience factors and mechanisms over stress-related dysfunction.

### 2.3. Concept of resilience

*Resilience* can be defined by the American Psychological Association Dictionary of Psychology (2023) as “a process and outcome of successfully adapting to difficult or challenging life experiences, especially through mental, emotional, and behavioral flexibility and adjustment to external and internal demands.” However, there is no consensus in the literature on the definition of this concept. We articulated our framework around Pascual-Leone and Bartres-Faz (2021)’s recent conceptualization, which emphasizes the active and dynamic aspects of resilience, and highlights the mechanisms and factors for positive adaptation to major sources of stress across the lifecourse (see also Kalisch et al., 2017, for a similar approach). Resilience depends on both pre-existing (resistance and reserve) and post-traumatic (compensation and coping) factors and mechanisms that mitigate the potential impact of a traumatic event on the brain at distinct post-traumatic stages and temporal scales (Pascual-Leone and Bartres-Faz, 2021).

#### 2.3.1. Mechanism of resistance in PTSD

*Resistance* can be defined as *psychological immunity*, namely, the ability to block the impact of a potentially stressful situation through different mechanisms (Nucifora et al., 2007; Karatsoreos and McEwen, 2011; Pascual-Leone and Bartres-Faz, 2021). These mechanisms may include cognitive control and ER. Cognitive control contributes to resistance insofar as effective cognitive control skills can block the occurrence of intrusions (Anderson and Green, 2001; Mary et al., 2020). Furthermore, symptoms such as a persistent negative emotional state can be prevented by means of an ER mechanism, as ER enables the up-and downregulation of positive and negative emotions according to the regulation objectives (McRae and Gross, 2020). As ER difficulties are associated with PTSD severity (Ehring and Quack, 2010), we can assume that an efficient ER mechanism is associated with a lower risk of manifesting PTSD symptoms. Resistance is therefore characterized by robust emotional and memory circuits to counter stress. Resistance contributes to resilience by blocking the impact of a potential traumatic event, while reserve contributes by diminishing the impact of a potential traumatic event (Pascual-Leone and Bartres-Faz, 2021).

#### 2.3.2. Mechanism of reserve in PTSD

With regard to the definition of the *Reserve* proposed by Pascual-Leone and Bartres-Faz (2021), reserve refers to “brain mechanisms, properties, and capacities that allow for behavior, cognition, and wellbeing to be better than expected, given the severity of the insult or disease” (p. 5). This concept should be relevant in all cases where the brain is injured, as it can limit the appearance of symptoms (Stern, 2002; Pascual-Leone and Bartres-Faz, 2021). Two aspects of reserve can be distinguished: *cognitive reserve* (CR) and *brain reserve* (BR) (Stern et al., 2020). CR is an *active* process. It can be either a *cognitive factor*, reflecting the daily activities in which a person engages and measured using sociobehavioral indices (e.g., education and leisure time activity), or a *cognitive mechanism* such as cognitive control (Medaglia et al., 2017), which can be quantified through functional neuroimaging (Stern, 2002; Stern et al., 2020). For instance, a recent prospective longitudinal study using functional MRI suggested that higher baseline anterior PFC, dorsal and medial frontal pole activity would limit the number of symptoms of PTSD that developed following the trauma (Kaldewaij et al., 2021). Reserve also refers to the BR, linked to the CR. BR refers to a *passive* process that depends on characteristics of the brain structure (e.g., regional brain size, synaptic and neuronal density) and can be measured by structural neuroimaging (Satz, 1993; Stern, 2002; Stern et al., 2020). The greater the BR, the longer the clinical deficit takes to manifest itself (Satz, 1993). However, if two patients have the same BR capacity, it is the patient with the highest CR who will be able to tolerate more brain damage before the onset of clinical symptoms (Stern, 2009). The concept of reserve is relevant to neuropsychiatric disorders (Barnett et al., 2006). With regard to PTSD, a smaller hippocampus and abnormalities in the amygdala are predisposing factors for psychological trauma, according to some studies (Gilbertson et al., 2002; Admon et al., 2013).

Resistance and reserve mechanisms provide prevention and protection in the event of exposure to a traumatic event. Once a traumatic event has occurred, two other processes come to the fore: compensation and coping.

#### 2.3.3. Compensation process

The *compensation* process, defined in Cabeza et al. (2018)’s consensus paper as “the cognition-enhancing recruitment of neural resources in response to relatively high cognitive demand” (p. 7), therefore serves to maintain cognitive performances to adapt and reduce the effects of stress generated by trauma. According to these authors, there are three types of compensation: compensation by upregulation, compensation by selection, and compensation by reorganization. Depending on the task demands, compensation by upregulation quantitatively increases pre-existing neural processes, resulting in improved cognitive performance. Compensation can also be achieved by selectively enhancing a specific cognitive process that enables the task to be achieved. Finally, compensation by reorganization involves the recruitment of alternative neural mechanisms, as the networks that are usually recruited are not available, owing to brain damage.

#### 2.3.4. Coping strategies

Coping strategies are put in place to maintain or restore wellbeing following a stressor (Pascual-Leone and Bartres-Faz, 2021). After exposure to traumatic situation, two behavioral coping strategies are frequently implemented: active coping strategies, and passive coping strategies such as avoidance (Thompson et al., 2018). Active coping strategies are behavioral and/or psychological strategies aimed at changing what directly concerns the stressor or how it is perceived, whereas avoidant coping involves activities and mental processes that do not directly address the stressor (e.g., alcohol use) (Wu et al., 2013; Thompson et al., 2018). The literature has consistently shown that active coping strategies are associated with greater resilience, while avoidant strategies are often regarded as maladaptive coping strategies (Linley and Joseph, 2004; Olff et al., 2005; Pineles et al., 2011; Wu et al., 2013; Thompson et al., 2018). Interestingly, active coping strategies correlate with activity in the left frontopolar cortex, whereas passive coping strategies are related to the activity of the anterior cingulate cortex (Santarnecchi et al., 2018).

Among the effective coping strategies that can be used, *cognitive reappraisal* allows way of thinking about a situation to be modified in order to influence the associated emotional response (McRae and Gross, 2020). This strategy involves an ER mechanism underpinned by cognitive control networks (Santarnecchi et al., 2018; Roeckner et al., 2021). Cognitive control mechanisms are activated to identify and resolve competition from irrelevant memories (Kuhl et al., 2007). Underpinned by the process of interference inhibition, the positive appraisal style is a key mechanism, protecting against the deleterious effects of stress (Kalisch et al., 2015). Coping strategies that activate frontal regions may be more beneficial because they allow for the reinforcement of control mechanisms, and thence better inhibitory abilities.

Pascual Leone’s work highlights the dynamic aspect of resilience, which depends on pre-and post-traumatic factors (Pascual-Leone and Bartres-Faz, 2021). Among these factors, musical practice and listening have often been studied as therapeutic tools (Bensimon et al., 2008; Carr et al., 2012; Ophir and Jacoby, 2020; Beck et al., 2021), but the interactions between musical experience and resilience remain complex. Indeed, professional musicians appear to be more exposed to mental health problems (Kenny et al., 2014; Niarchou et al., 2021) but as Gustavson et al. (2021) recently pointed out, it’s quite possible that musicians use music as a means of managing these problems (the analogy is made with anti-depression medication, which is more widely used by depressed people precisely *because* it’s a treatment). These associations between music and mental health need to be untangled, which is why we need to look more closely at how musical experience might interact with the resilience model.

## 3. Discussion: music as a resilience factor against PTSD?

Methodologically rigorous studies are still needed but work suggesting a reduction in the intensity of PTSD symptoms through musical interventions offers promising prospects (Landis-Shack et al., 2017). A recent review of the literature by Pant et al. (2022) identified key contributions of music listening and playing for people with PTSD. These included reductions in stress, anxiety, and PTSD symptoms. A strong case was made for building on the potentially therapeutic contribution of music and sound interventions to PTSD symptoms after hospitalization in an intensive care unit. For example, the beneficial role of musical activity in restoring the imbalance caused by the dysfunctional HPA axis feedback loop in PTSD is developed. Interestingly, this rebalancing could result in lower cortisol levels thanks to musical activity. In this review, music was not regarded as a protective or resilience factor, but rather as a relevant medium for the treatment of patients with PTSD. Numerous other studies consider music primarily as a coping strategy (Garrido et al., 2015; Story and Beck, 2017; Ophir and Jacoby, 2020) without considering that a targeted use of music could be proposed as a preventive measure to promote resilience.

Our present conceptual analysis is more in line with research underscoring the importance of considering resilience as a dynamic process involving both pre-and post-traumatic mechanisms (Kalisch et al., 2015, 2017; Pascual-Leone and Bartres-Faz, 2021). We therefore suggest that there needs to be a paradigm shift, in order to explore how musical experience may contribute to resilience across the lifecourse by stimulating reserve and resistance mechanisms.

### 3.1. How musical experience may interact with reserve and resistance

**Figure 1:**
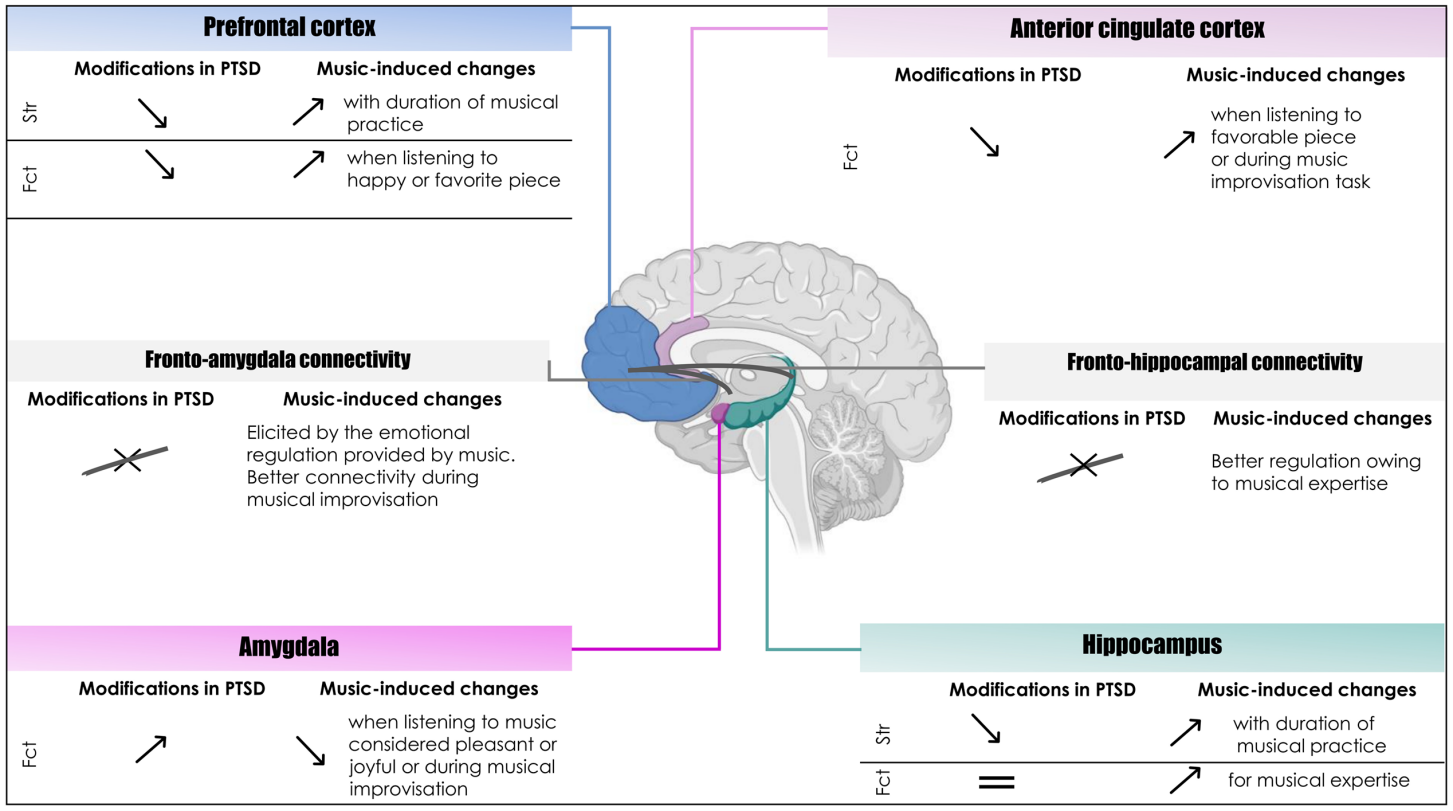
Structural and functional modifications associated with PTSD in relation to the structural and functional modifications induced by playing or listening to music. Upward arrows indicate either an increase in gray-matter volume (“Str” for structural modifications) or hyperactivation (“Fct” for functional modifications). Downward arrows indicate either a reduction in gray-matter volume (for structural modifications) or hypoactivation (for functional modifications). Equal sign: no change. Crossed line: impaired connectivity. Created in BioRender.com.

Regarding the potential impact of musical experience during the pre-trauma period, there has been scant research on the effects of playing or listening to music on the mechanisms of resistance. Concerning reserve, one study has suggested that biological and environmental factors contribute to the constitution of a *resilience reserve* comparable to Stern et al. (2020)’s CR, protecting against the development of PTSD (Rakesh et al., 2019). The potential benefits of musical experience in terms of building CR and BR to protect against PTSD symptoms were not explored in their work. A systematic review of pre-incident preparation to prevent the development of PTSD identified interventions including attention training, heartrate-based biofeedback, and a psychoeducational program (Bisson et al., 2021). None of these studies explored the potential of music as a protective factor. Music has so far been assumed to alleviate the symptoms of PTSD (Hernandez-Ruiz, 2005; Carr et al., 2012; Pezzin et al., 2018; Beck et al., 2021; Pant et al., 2022), but not to protect against the development of PTSD. Given the strong neuroplasticity that music induces (Gaser and Schlaug, 2003; Lappe et al., 2008; Stewart, 2008; Herholz and Zatorre, 2012), we can surmise that it contributes to the building of resistance and reserve (Herholz et al., 2013; Baird and Samson, 2015; Andrews et al., 2021; Böttcher et al., 2022), as the brain areas that are functionally and anatomically affected in PTSD are precisely those that are activated during listening and musical practice (Figure 1; Table 1 in Supplementary material). More specifically, music leads to structural and functional modifications in a number of brain areas, including the motor cortex, auditory cortex, frontal cortex, anterior cingulate cortex, hippocampus, and amygdala (Stewart, 2008; Bermudez et al., 2009; Groussard et al., 2010; Rodrigues et al., 2010; Koelsch et al., 2013; Fauvel et al., 2014). The PFC, anterior cingulate cortex, amygdala, and hippocampus are also known to be impacted by PTSD. The present conceptual analysis explores the hypothesis that musical experience can be a pretraumatic resilience factor. Playing and listening to music may promote the constitution of resistance and reserve, protecting against the ER difficulties and cognitive control impairments associated with PTSD.

### 3.2. How music can protect against PTSD by triggering the emotion regulation mechanism

*Proposal 1:* Playing or listening to music may contribute to the building of resistance and reserve (CR and BR), thereby avoiding or limiting emotion regulation problems in PTSD.

To our knowledge, no study has yet considered music as a means of averting ER difficulties in PTSD, even though ER enabled by music listening and practice can be regarded as a protective mechanism. Music is well known to evoke strong emotions (Juslin, 2013; Koelsch et al., 2013; Koelsch, 2015), and listening to music is frequently used for ER in everyday life (Thoma et al., 2012; Saarikallio et al., 2013; Cook et al., 2019). In 2011, Londsale and North identified several factors that explain why we listen to music, including *negative mood management*, used to reduce negative feelings, and *positive mood management*, used to increase positive feelings (Lonsdale and North, 2011). These two factors are particularly important when an individual’s emotional state is disturbed, as in the case of prolonged stress. The way music was recently used during the COVID-19 pandemic is a good illustration of its potential to modify emotional states. For example, during the lockdown in Germany in Spring 2020, Roese and Merrill (2021) observed that before the pandemic, music was mostly used to enhance wellbeing, whereas during the lockdown, music was used more to overcome loneliness. Using an online survey, Hennessy et al. (2021) found a positive correlation between the use of music listening for mood regulation and wellbeing, illustrating how musical engagement can be adapted to the general context and evolves in tandem with emotional state. The link between ER and reserve was explored in a study conducted among individuals with late-life depression (Huang et al., 2019). This showed that individuals with better CR had fewer ER difficulties than those with poorer CR. Like depressed older people, individuals with PTSD exhibit ER difficulties, which contribute to the deterioration of their emotional state (Ehring and Quack, 2010; McLean and Foa, 2017). We can therefore hypothesize that activities that reduce ER difficulties, such as listening to music and musical practice, contribute to the building of a protective reserve that prevents or limits the deterioration of the emotional state of people exposed to trauma.

The preventive use of music for ER is underpinned by mechanisms that may contribute to resistance and reserve, thereby blocking or limiting structural and functional damage to key regions involved in ER in PTSD (Liberzon, 2006; Shin, 2006; Harnett et al., 2020). Most studies investigating emotion with music have done so from a functional point of view (Koelsch et al., 2006; Koelsch, 2014).

The neural activation patterns underlying ER involve an interaction between frontal regions associated with cognitive control and the amygdala (Banks et al., 2007; Koenigs and Grafman, 2009; Andrewes and Jenkins, 2019). Studies have reported that these patterns can be activated by playing or listening to music (Moore, 2013; Hou et al., 2017). A systematic review of studies examining the neural effects of playing or listening to music, mainly using fMRI and PET, reported that listening to pleasant or happy music activates the anterior cingulate cortex and prefrontal regions such as the orbitofrontal cortex, and reduces activation of the amygdala (Blood and Zatorre, 2001; Moore, 2013), whereas listening to unpleasant or sad music increases amygdala activation (Koelsch et al., 2006). Thus, activation or deactivation of the amygdala depends on the type of musical experience (Moore, 2013). As part of pre-trauma training to promote ER and in line with Moore’s recommendations, the musical stimuli chosen in musical intervention should not be dissonant, unpleasant, unexpected, in a minor key, listened to with the eyes closed, or involve frequent chord changes as these stimuli lead to an increase in amygdala activity when the desired goal is a decrease in amygdala activity (Moore, 2013). To deactivate the amygdala, pre-trauma training should be based on listening to pleasant music or practicing musical improvisation according to Moore’s guidelines. Moreover, an fMRI study revealed better connectivity between frontal regions and the left amygdala during musical improvisation (Liu et al., 2012), while Banks et al. (2007) highlighted the importance of fronto-amygdala connectivity during ER (Banks et al., 2007). These differences in activation patterns according to musical experience point to opportunities to promote ER. In the context of PTSD, playing or listening to music could promote the activation of prefrontal regions and decrease the activation of the amygdala, thereby contributing to CR and resistance to protect against the functional impairments brought about by PTSD (Figure 1).

In PTSD, structural damage takes the form of a reduction in the volume of the prefrontal regions and anterior cingulate cortex. To our knowledge, no study has yet demonstrated structural changes in the anterior cingulate cortex as a result of playing or listening to music. However, interesting results suggest that gray-matter volumes in the right superior and middle frontal cortices gradually change with musical expertise (Groussard et al., 2014), strongly supporting the argument that musical experience contributes to BR.

There are strong interactions between emotions and cognition (Pessoa, 2010; Eustache et al., 2017; Reisenzein, 2019), and ER mechanisms also depend on control mechanisms via the prefrontal regions, hence the value of considering the positive influence of playing or listening to music on control mechanisms.

### 3.3. How music can protect against PTSD by triggering the cognitive control mechanism

*Proposal 2:* Playing or listening to music may contribute to the building of resistance and reserve (CR and BR) in order to block or limit intrusions in PTSD.

Authors have recently hypothesized that intrusions may arise not only from a failure of memory, but from a deficit in the control system that fails to inhibit traumatic memories, suggesting that PTSD is a forgetting disorder (Mary et al., 2020). It would therefore be useful to ask whether playing or listening to music also influences the cognitive control mechanisms that allow for active forgetting.

Individual differences in inhibiting unwanted memories can be explained by differences in executive control (Levy and Anderson, 2008), and although authors have yet to establish whether executive functioning disorders are a cause or a consequence of PTSD, executive functions and inhibition are particularly impaired in this disorder (Tapia et al., 2007; Aupperle et al., 2012; Polak et al., 2012; Quinones et al., 2020; Bisson et al., 2021). Inhibition seems to be the executive function most closely correlated with the severity of PTSD symptoms, according to studies carried out among veterans (Swick et al., 2012; DeGutis et al., 2015). Interestingly, the literature suggests that musicians exhibit better executive performances than nonmusicians (Zuk et al., 2014; Groussard et al., 2020). More specifically, musical practice is thought to have an impact on inhibition abilities at all ages of life (Moreno et al., 2011; Slater et al., 2017; Jaschke et al., 2018). Children aged 4–6 years who had participated in a music-based 20-day program performed better on a Go/No-go task measuring inhibition than those who had received a computerized visual arts intervention (Moreno et al., 2011). Moreover, in a longitudinal study of 147 primary-school children, scores on inhibition and planning tests improved over time in the music intervention groups, whereas no significant improvements were reported for the visual arts group and the control group without art (Jaschke et al., 2018). These results were also observed in adults by Slater et al. (2017), who compared adult nonmusicians, vocalists and percussionists on inhibitory control abilities. Results revealed that percussionists performed better on inhibitory control than both nonmusicians and vocalists. The use of music could help strengthen control mechanisms, and thereby limit intrusions. Thus, the effects of music on executive functioning could contribute to the building of resistance and CR to protect against the effects of PTSD.

Musical experience is also linked to anatomical and functional changes that may contribute to resistance and reserve, again to protect against the effects of PTSD. On the anatomical level, concerning the left hippocampus and right superior and middle frontal cortices, gray-matter volume gradually changes with musical expertise (Groussard et al., 2014). This can be explained by the time needed to master an instrument, in addition to coordinating with other musicians in order to play together. Concerning the functional effects of musical practice, Fauvel et al. (2014) observed better connectivity at rest in musicians versus nonmusicians, and activation of areas extending from subcortical regions to frontal regions, via the sensorimotor cortex. Interestingly, an fMRI study showed that the strength of top-down modulation of the hippocampus by the left inferior frontal gyrus was greater in musicians than in nonmusicians, suggesting that musical expertise allows for better cognitive control (Gagnepain et al., 2017a). Finally, studies have indicated that listening to music activates different regions including frontal regions (Koelsch, 2014; Ding et al., 2019). These results point to the potential of music to activate cognitive control mechanisms that are precisely deficient in PTSD.

**Figure 2:**
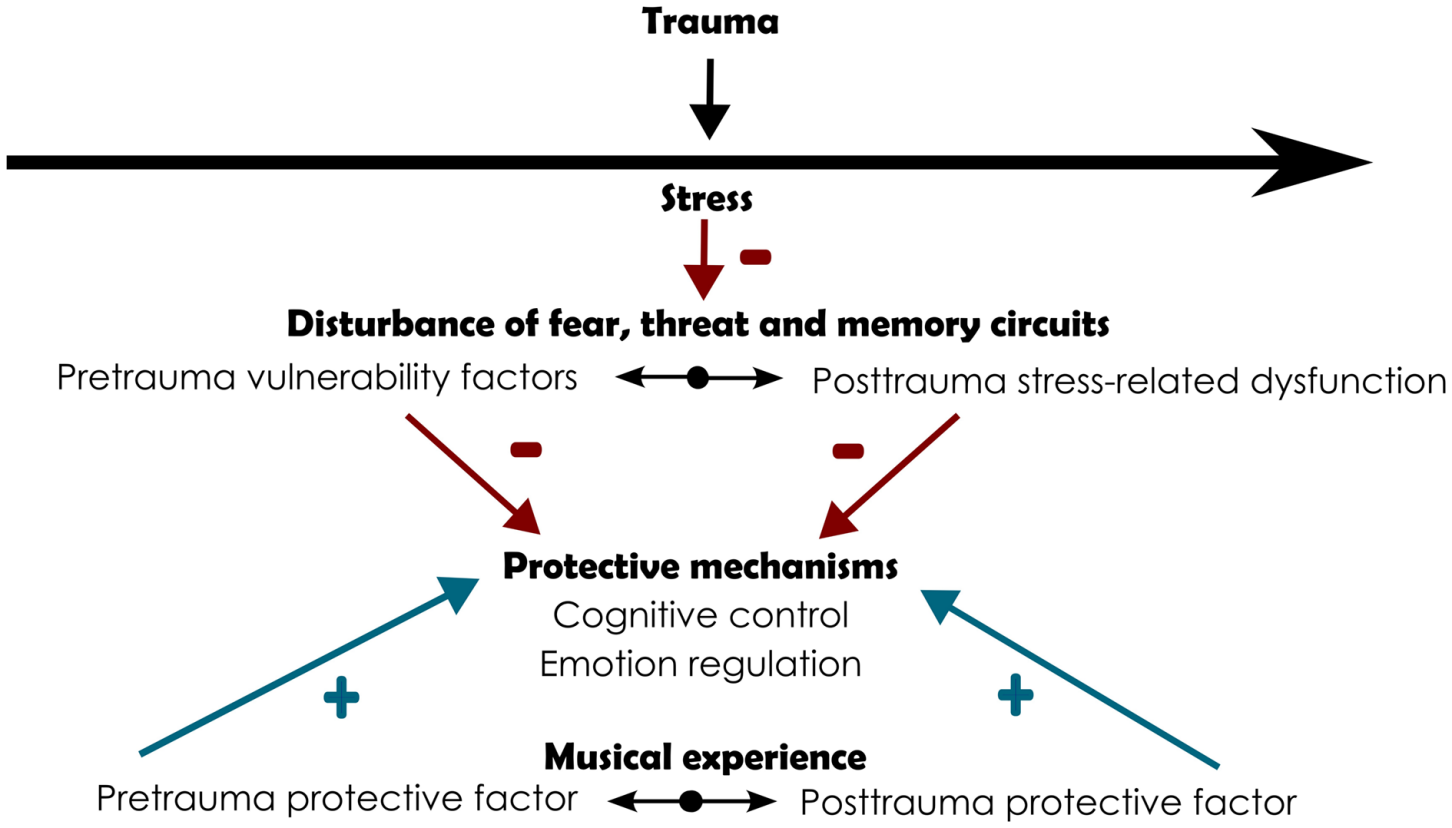
Contributions of musical experience to resilience through the stimulation of protective mechanisms in pre- and post-trauma periods. Blue arrows with plus signs: deleterious effects. Red arrows with minus signs: strengthening effect.

Resilience is a dynamic process that takes place across the lifecourse. ER and cognitive control mechanisms can be called upon before trauma for building resistance and reserve and after trauma to cope with the deleterious consequences of stress (Figure 2). We can therefore assume that the earlier these mechanisms are called upon by playing and listening to music, the greater the resilience individuals have to cope with emotional dysregulation and intrusions.

## 4. Concluding remarks

Interventional studies have reported the value of listening to music and musical practice after trauma (Landis-Shack et al., 2017; Story and Beck, 2017; Ophir and Jacoby, 2020; Pant et al., 2022), but the arguments set out in this analysis lead us to believe that the value of music may be even more pronounced in people who had a special relationship with music before being exposed to trauma. Pre-traumatic musical experience may well be a resilience factor, given that it influences mood, cognitive and cerebral spheres.

Regarding mood, music enhances ER, and thus provides more resources to overcome PTSD-related negative emotional state (Moore, 2013; Pant et al., 2022). On the cognitive level, several studies have shown that people who have received musical training have better executive control than those who have not received any musical training (Zuk et al., 2014; Groussard et al., 2020). Thus, people who have played or listened to music may be more resilient than those who have no such musical experience. In the same idea, at cerebral level, playing or listening to music induces both functional and structural plasticity (Jäncke, 2009; Groussard et al., 2010; Herholz and Zatorre, 2012; Fauvel et al., 2014; Schlaug, 2015), particularly in the frontal regions, hippocampus, and amygdala, structures that appear to be affected in PTSD.

Consequently, playing and listening to music seem to be activities that can promote the building of resistance and reserve (Herholz et al., 2013; Andrews et al., 2021; Böttcher et al., 2022). Building up good CR and BR across the lifecourse delays the clinical manifestation of many neurological pathologies (Barnett et al., 2006; Baird and Samson, 2015). Music may therefore protect against the emergence of PTSD symptoms such as such emotional dysregulation and intrusions, although no study has yet directly investigated the impact of prior musical experience (playing/listening) in individuals who have experienced trauma.

### 4.1. Research perspectives

More work is needed to understand the underlying mechanisms and the potential protective effects of music. In order to explore music as a pre-traumatic resilience factor and complementing the elements detailed in this conceptual analysis, it might be relevant to study how musical experience prior to traumatic exposure might engage the neuroendocrine system to protect against the development of PTSD. In addition, to study whether and how musical experience acquired prior to potential trauma can protect against the onset of PTSD, different elements will need to be investigated.

First, it could be interesting to consider playing or listening to music as a proxy measure of CR. To measure BR, neuroimaging techniques such as anatomical MRI could be used to observe the BR of musicians versus nonmusicians with PTSD. Brain synchrony could be another imaging data analysis methodology for studying the potential protective effects of music in the context of PTSD.

Second, to limit the sources of interindividual variation, it would be useful to select participants with the same traumatic origin and take into account as many covariates as possible, such as age and education level.

Third, consistent with recent literature emphasizing the need for prospective longitudinal studies to identify resilience factors (Kalisch et al., 2017; Kaldewaij et al., 2021), research on resilience in relation to PTSD could also be carried out prior to any traumatic event, to ascertain the value of using musical training to protect against the potential emergence of PTSD.

### 4.2. Clinical perspectives

Beyond research perspectives, our conceptual analysis also opens the way to clinical implications. Indeed, it seems relevant to consider the pre-traumatic musical experience as a factor blocking or limiting the development of PTSD. In concrete terms, the clinical application could be to study how to structure musical stimuli to build pre-trauma training that promotes emotional regulation and cognitive control, aimed at populations at risk of developing PTSD such as healthcare workers, police officers, and military staffs.

To conclude, this still understudied field of research on resilience mechanisms is therefore highly promising, from both a fundamental and a public health point of view.

## Author contributions

All authors listed have made a substantial, direct, and intellectual contribution to the work and approved it for publication.

## Conflict of interest

The authors declare that the research was conducted in the absence of any commercial or financial relationships that could be construed as a potential conflict of interest.

## Publisher’s note

All claims expressed in this article are solely those of the authors and do not necessarily represent those of their affiliated organizations, or those of the publisher, the editors and the reviewers. Any product that may be evaluated in this article, or claim that may be made by its manufacturer, is not guaranteed or endorsed by the publisher.
