# Supplementary material for: Musical experience prior to traumatic exposure as a resilience factor: a conceptual analysis
Source: Front Psychol. 2023 Jul 27;14:1220489. doi: 10.3389/fpsyg.2023.1220489 (PMC10436084; doi:10.3389/fpsyg.2023.1220489)
Supplement: Supplementary file 1 [file Table_1.pdf]

Supplementary material

Table 1. Structural and functional modifications associated with PTSD in relation to the structural and functional modifications induced by playing or listening to music.

	Modifications in PTSD		Music-induced changes	
	Structural	Functional	Structural	Functional
Prefrontal cortex	Atrophy (Harnett et al., 2020; Shin, 2006)	Hypoactivation (Harnett et al., 2020; Shin, 2006)	Gray matter volume of right superior and middle frontal cortex increases **with duration of musical practice** (Groussard, 2014)	Activation of the prefrontal cortex **when listening to happy or favorite piece** (Moore, 2013) Supplementary activations in the medial frontal gyrus induced by musical expertise (Groussard, 2010)
Hippocampus	Atrophy (Geuze et al., 2005; Logue et al., 2018; Postel et al., 2021; Woon & Hedges, 2008; Yehuda & LeDoux, 2007)	No clear pattern (Geuze et al., 2005; Yehuda & LeDoux, 2007)	Gray matter volume of the left hippocampus increases **with duration of musical practice** (Groussard, 2014)	**Supplementary activations induced by musical expertise** (Groussard, 2010)
Anterior cingulate cortex		Hypoactivation (Dossi et al., 2020; Fitzgerald et al., 2018)		Activation of the left ACC **when listening to favorable piece and** activation of the right ACC **during music improvisation task** (Blood & Zatorre, 2001; Moore, 2013)
Amygdala		Hyperactivation (Harnett et al., 2020; Hayes et al., 2012; Shin, 2006)		**Decreased** amygdala activation **when listening to music considered pleasant or joyful and during musical improvisation** (Blood & Zatorre, 2001; Moore, 2013)
Fronto-hippocampal connectivity	Impaired connectivity (Leone et al., 2022; Mary et al., 2020)		**Better top-down modulation owing to musical expertise** (Gagnepain, 2017a)	
Fronto-amygdala connectivity	Impaired connectivity (Andrewes, 2019)		Elicited by the emotional regulation provided by music. **Better medial prefrontal-amygdala connectivity during musical improvisation** (Liu et al, 2012)	
